# Novel application of an optical inspection system to determine the freshness of *Scomber japonicus* (mackerel) stored at a low temperature

**DOI:** 10.1007/s10068-019-00639-z

**Published:** 2019-08-08

**Authors:** Jeong-Wook Choi, Myung-Kee Jang, Chang-Wook Hong, Ju-Woon Lee, Jae-Hyuk Choi, Koth-Bong-Woo-Ri Kim, Xiaotong Xu, Dong-Hyun Ahn, Min-kyeong Lee, Taek Jeong Nam

**Affiliations:** 1 Institute of Fisheries Sciences, Pukyong National University, Busan, 46041 Republic of Korea; 2 Devicenet Research Institute, Anyang, Gyeonggi-do 14056 Republic of Korea; 3 Department of Food Science and Technology/Institute of Food Science, Pukyong National University, Busan, 48513 Republic of Korea; 4 Department of Food Science and Nutrition, Pukyong National University, 45, Yongso-ro, Nam-Gu, Busan, 48513 Republic of Korea

**Keywords:** Mackerel, Freshness, Nondestructive, Quality, Optical inspection system

## Abstract

This study evaluated the use of an optical inspection system (OIS) to determine the freshness of mackerel (*Scomber japonicus*). The correlations between the light reflection intensity (LRI) of mackerel eyes (determined using an OIS) and the volatile basic nitrogen content (VBN) and K-value were analyzed. After unloading at the harbor, the mackerel were stored at 4 °C for 9 days and the VBN, K-value, and LRI were determined at 3-day intervals. During storage, the LRI, VBN, and K-value all increased. Furthermore, the LRI was correlated with the K-value and VBN. Therefore, although the LRI cannot be applied as an absolute standard for evaluating freshness, the LRI using an OIS is a suitable nondestructive method for evaluating freshness for quality and risk management in the processing industry when handling large numbers of fish.

## Introduction

Freshness of fish is the most important aspect because it affects taste, texture and safety. Therefore, the priority value in purchasing fish is freshness. (Huang et al., 2011). However, consumers often cannot obtain objective information about the freshness, quality, and shelf life of mackerel during the sales process, and producers are unable to manage the quality and risk for all of the mackerel handled. Therefore, an improved freshness evaluation method is required to solve this problem and to increase consumer consumption of fish.

The quality and freshness of fish can be measured by using the levels of trimethylamine (TMA), volatile basic nitrogen (VBN), and thiobarbituric acid reactive substances (TBARS), and the K-value (Chang et al., 1998; Kuda et al., 2002). These measurement methods have been evaluated many times (Cheng et al., 2015). However, it takes considerable time to analyze the freshness and quality of fish during distribution, especially examining every fish because fish tissue is required for analysis.

Nondestructive inspection methods have occasionally been used to monitor and evaluate food quality (Daugaard et al., 2010; Huang et al., 2013; Paluchowski et al., 2016; Uddin et al., 2006), but there are no reports using optical inspection methods to measure the freshness of fish, especially the light reflection intensity (LRI) of eyes.

In this study, we used changes in the transparency of the eyes to measure freshness. Eye transparency and physicochemical analyses were conducted on mackerel stored at 4 °C. Then, the correlations between the LRI and physicochemical results were evaluated to assess whether they were appropriate for measuring freshness.

## Materials and methods

### Sample preparation

Mackerel (*Scomber japonicus*) were purchased from a wholesale fishery market (Busan, Korea) just after being unloaded from a fishing boat. The mackerel samples were tagged with a numbered label on their tailfin and stored in a refrigerator at 4 °C.

### Optical inspection

#### The optical inspection system (OIS)

The OIS system consisted of an optical camera (Basler Ace A2000-50gc color CCD; Basler, Ahrensburg, Germany), lens (EDMUND 8.5 mm/F1.3, C-Mount; Edmund, Barrington, NJ, USA), and light source (white LED 20 W with a dome fixture; LFINE, Incheon, Korea). Images of the eyes were captured at 50 frames/s by a Gigabit Ethernet with Lab-view I-MAQ-dx Library (VDM; National Instruments, Austin, TX, USA), and the red–green–blue color (RGB) format intensity of each eye was processed in a 64-bit industrial PC environment.

#### Measurement of the eye reflection intensity

**Fig. 1:**
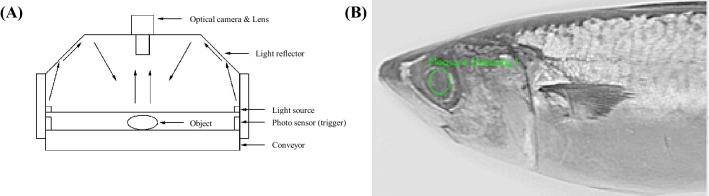
(**A**) The optical inspection system (OIS) and (**B**) a photograph used for automatic eye recognition with the OIS (application of blue and green removal filter)

The LRI was measured with the OIS. The mackerel were put on a conveyer and passed through the optical system (Fig. 1A). As each mackerel moved under the optical camera and 11,700 lx light source, its whole body was captured by the optical camera (2000 μs exposure) and in order to uniformly illuminate the mackerel, the light reflector was used to induce dispersion of light. A program automatically selected the eye region (Fig. 1B) in mackerel picture using optical camera. Then, the RGB value of the eye image was measured according to the RGB color classification. In the analysis, only the red value was used because it had the closest relationship to the changes in freshness. The analyzed values were saved as the LRI after calculating the average and deviation.

### Chemical analysis

#### VBN

The VBN content was determined by the Conway method following the guidelines of the Ministry of Food and Drug Safety (MFDS, 2016). Briefly, a 10 g mackerel sample and 50 mL of deionized distilled water (DDW) were put in a glass beaker and homogenized with a homogenizer (AM-7; Nihon Seiki Kaisha, Tokyo, Japan). The homogenate was stirred gently in a shaker for 10 min. After shaking, the homogenate was allowed to stabilize for 5 min at room temperature (RT). This process was repeated three times. The homogenate was centrifuged at 2000×*g* for 10 min and the supernatant was filtered through Whatman No. 4 filter paper. The pH of the filtrate was adjusted to 4.0 by adding 5% H_2_SO_4_ and the volume was made up to 100 mL with DDW. Then, 1.0 mL of 0.01 N H_2_SO_4_ was put in the inner space of the Conway unit and 1.0 mL of saturated K_2_CO_3_ and the sample were added to the outer space of the Conway unit. The Conway unit was sealed immediately and incubated at 25 °C, with 30% humidity, for 1 h. After incubation, one drop of Brunswik reagent (0.2 g of methyl red and 0.1 g of methylene blue in 300 mL of ethyl alcohol) was added to the inner space of the Conway unit. The solution was titrated using 0.01 N NaOH and the VBN content was calculated using the following equation:

$${\text{VBN}}\,\left( {{\text{mg}}/100\,{\text{g}}} \right) = 0.14 \, \times \, \left[ {\left( {A {-} B} \right) \times F} \right]/W \times 100 \, \times D,$$

where *A* is the volume (mL) of 0.01 N NaOH used for sample titration, *B* is the volume (mL) of 0.01 N NaOH used for blank titration, *F* is the factor of 0.01 N NaOH, *W* is the sample weight (g), and *D* is the dilution factor.

#### K-value

First, a 10.0 g mackerel sample was homogenated with 30 mL of chilled 10% perchloric acid in a glass beaker and allowed to stabilize for 30 min at RT. The homogenate was centrifuged at 2000×*g* for 20 min and the supernatant was collected. The precipitate was resuspended in chilled 10% perchloric acid and centrifuged under the same conditions. The supernatants from two extractions were pooled and filtered with Whatman No. 4 filter paper. The filtrate was neutralized with chilled 5 M KOH to pH 6.5 and the final volume adjusted to 100 mL using DDW. Then, the resulting solution was filtered through a 0.45 μm membrane (Cat. no. 4408; PALL Corp., Ann Arbor, MI, USA), and 10.0 μL of the filtrate were injected into a high-performance liquid chromatography (HPLC) instrument (1100 Series; Agilent Technologies, Palo Alto, CA, USA) to analyze nucleic acid-related compounds [NARCs; adenosine triphosphate (ATP), adenosine diphosphate (ADP), adenosine monophosphate (AMP), inosine monophosphate (IMP), inosine, (HxR), and hypoxanthine (Hx)]. The chemical reagents used as NARC standards were purchased from Sigma Aldrich (St. Louis, MO, USA).

The analytical conditions for HPLC were as follows: ultraviolet (UV) detection at 254 nm, the range of absorbed dose (absorbance units full scale; AUF) was 0.5, µBondapak C_18_ column (3.9 mm i.d. × 300 mm; Waters, Milford, MA, USA), column chamber temperature 40 °C, flow rate 2.0 mL/min, and mobile phase 1% tri-ethylamine (pH 6.5) adjusted with 10% H_3_PO_4_. The K-value was calculated using the following equation:

$${\text{K (}}\% )= \frac{{ ( {\text{HxR + Hx)}}}}{\text{ATP + ADP + AMP + IMP + HxR + Hx}} \times 100.$$

### Statistical analysis

Values are presented as mean ± standard deviation (SD). The data were analyzed with SPSS ver. 18.0 (SPSS Inc., Chicago, IL, USA) using a one-way analysis of variance followed by Duncan’s multiple range test. Furthermore, Pearson correlation analysis was used to assess the correlation of freshness between the LRI and chemical analysis results (VBN, K-value). *P*-values < 0.05 were considered to indicate statistical significance.

## Results and discussion

### Evaluation of freshness with chemical analyses

**Fig. 2:**
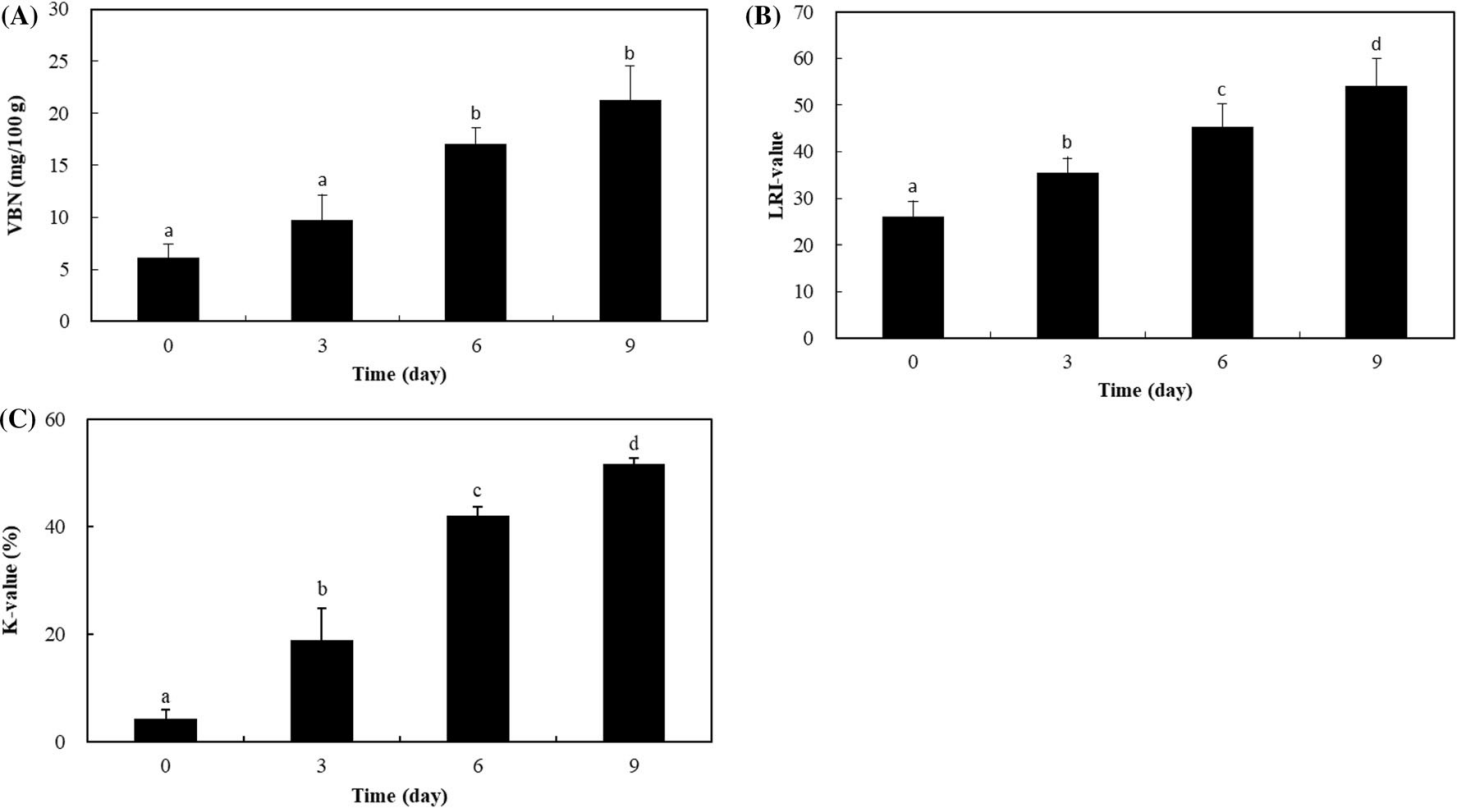
(**A**) VBN content, (**B**) K-value, and (**C**) LRI of mackerel during storage at 4 °C for 9 days. The values are the mean ± SD. Different lowercase letters indicate significant differences at *p* < 0.05

When the mackerel were stored at 4 °C, the VBN content increased rapidly during storage from 6.11 mg/100 g at day 0 to 21.26 mg/100 g after 9 days (Fig. 2A). Generally, VBN content is 5–10 mg/100 g when the fresh fish, in the moderately fresh 15–25 mg/100 g; when the fish started to spoil, it increased to 30–40 mg/100 g, exceeding 50 mg/100 g when it was spoiled (Tsai et al., 2018).

The K-value is an important index of fish freshness based on nucleotide degradation. During autolysis in fish, ATP gradually breaks down into ADP, AMP, IMP, HxR, and Hx as a result of enzymatic and microbial activity (Lowe et al., 1993). As degradation progresses, the HxR and Hx contents increase markedly (Hamada-Sato et al., 2005). When determining freshness using the K-value, fresh fish should have a value < 10%, sashimi 10–20%, moderately fresh 20–50%, raw material for processing 35–60%, and spoiled material > 60% (Huang et al., 2015). The K-value of mackerel stored at 4 °C increased rapidly during storage (Fig. 2B), from 4.21% initially to 51.71% (about 12.28 times) after 9 days.

### Changes in LRI

During storage at 4 °C, the LRI of mackerel increased with the length of storage (Fig. 2C), from 26.04 at day 0 to 37.5 (1.4 times), 40.62 (1.5 times), and 44.55 (1.7 times) at days 3, 6, and 9, respectively. Initially, the mackerel eyes were transparent and the LRI was low. During storage, the LRI increased gradually. It is thought that the light-reflecting intensity of the fish eyes was inhibited by the change in turbidity of the eye lens. The LRI was highly correlated with the K-value (r = 0.906) and VBN content (r = 0.911) (Table 1). However, it was impossible to estimate the K-value or VBN content in reverse based on the LRI only. Consequently, it is clear that the LRI is of limited value for evaluating freshness. Nevertheless, the LRI can be used to identify the freshness of fish to manage potential risks, and it has the advantage of being a nondestructive method that can potentially be applied when processing large numbers of mackerel.

**Table 1:** The Pearson correlations between the LRI and the VBN content and K-value in mackerel during storage at 4 °C for 9 days

	VBN content	K-value
LRI	0.911^a^	0.906^b^

Values are the mean ± SD

Different lowercase letters indicate significant differences at *p* < 0.05
